# Microbial hexuronate catabolism in biotechnology

**DOI:** 10.1186/s13568-019-0737-1

**Published:** 2019-01-30

**Authors:** Joosu Kuivanen, Alessandra Biz, Peter Richard

**Affiliations:** 1 VTT Technical Research Centre of Finland Ltd, PO box 1000, 02044 VTT Espoo, Finland; 2 Department of Chemical Engineering and Applied Chemistry, University of Toronto, 200 College st, Toronto, ON M5S 3E5 Canada

**Keywords:** d-Galacturonate, d-Glucuronate, Pectin

## Abstract

The most abundant hexuronate in plant biomass is d-galacturonate. d-Galacturonate is the main constituent of pectin. Pectin-rich biomass is abundantly available as sugar beet pulp or citrus processing waste and is currently mainly used as cattle feed. Other naturally occurring hexuronates are d-glucuronate, l-guluronate, d-mannuronate and l-iduronate. d-Glucuronate is a constituent of the plant cell wall polysaccharide glucuronoxylan and of the algal polysaccharide ulvan. Ulvan also contains l-iduronate. l-Guluronate and d-mannuronate are the monomers of alginate. These raw materials have the potential to be used as raw material in biotechnology-based production of fuels or chemicals. In this communication, we will review the microbial pathways related to these hexuronates and their potential use in biotechnology.

## Introduction

d-Galacturonate (d-galUA) is the most abundant hexuronic acid. It is the main monomer of pectin. Pectin-rich biomass is an important raw material but it has not attracted much attention. Some examples of pectin-rich biomass are sugar beet pulp (SBP), apple pomace and citrus processing waste (CPW). The potential use of this biomass for the production of fuels and chemicals has been reviewed in the past (Richard and Hilditch 2009; Edwards and Doran-Peterson 2012). The current communication covers new developments.

The worldwide production of sugar beet is about 250 million tons per year. From one ton of beet, about 150 kg of sugar and about 210 kg of pressed beet pulp are produced. The pressed beet pulp has a dry matter content of about 20%. Pressed beet pulp is often ensiled and used as cattle feed. The d-galUA content of the dry matter is about 21% (Micard et al. 1996). Citrus fruit production worldwide is about 115 million tons per year; however only about a third is processed in citrus juice factories, where about 50–60% of the fruit is CPW. CPW has about 20% dry matter, of which 40% is pectin. Unlike the pectin from SBP, CPW pectin can be used as a food ingredient for gelling purposes. However, the market for food pectin is much lower than the amount of pectin available from CPW.

d-Glucuronate (d-glcUA) is a constituent of the plant cell wall polysaccharide glucuronoxylan (Reis et al. 1994) and of the algal polysaccharide ulvan (Lahaye and Robic 2007). In addition, d-glcUA can be produced through biochemical routes from d-glucose via the *myo*-inositol oxidation pathway (Moon et al. 2009), which increases the relevance of d-glcUA-converting enzymes from the point of view of biomass processing.

## Pectin structure and enzymatic hydrolysis

**Fig. 1:**
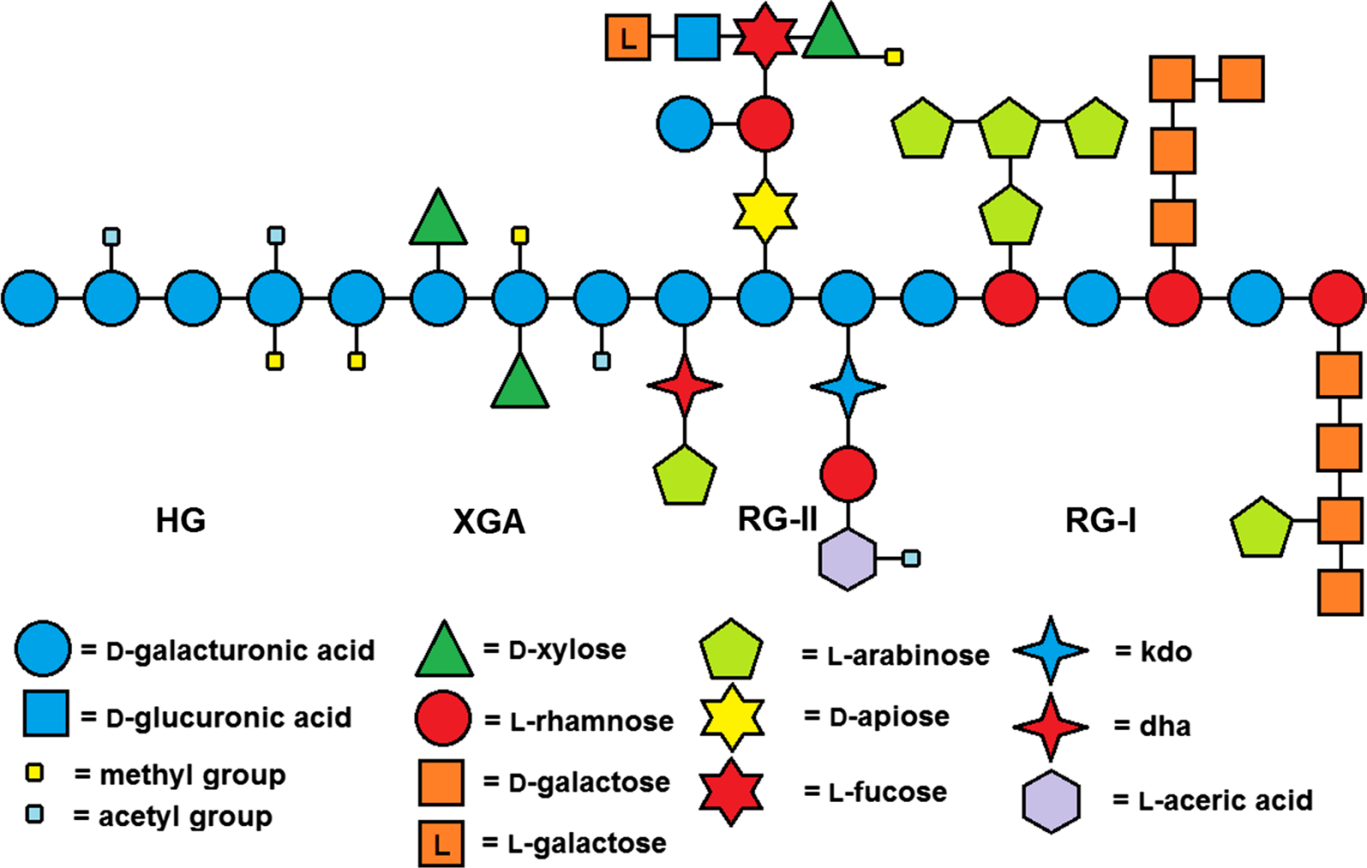
Structure of the pectic polymers homogalacturonan (HG), xylogalacturonan (XGA) rhamnogalacturonan II (RG-II) and rhamnogalacturonan I (RG-I). Figure from (Kuivanen 2015)

Pectins are a group of complex and flexible polymers that are found in plant biomass. They are abundant around growing or dividing plant cells and soft tissues such as fruit peels. The main monomer d-galUA is a common feature in all the pectic polymers, accounting for about 70% of the overall composition of pectin monomers (Mohnen 2008). Chemical structures of the pectin polymers can be divided into homogalacturonan (HG), the chemically more complex rhamnogalacturonan I (RG-I) and substituted HGs such as rhamnogalacturonan II (RG-2) (Fig. 1). HG is also referred to as smooth pectin, whereas RG-I and substituted HGs are referred to as hairy pectin.

HG consists of α-1,4-linked d-galUA units which are partially acetylated and methylesterified (Mohnen 2008). Accounting for about 65% of pectic polysaccharides, HG is the most common pectin type (Mohnen 2008). RG-I accounts for about 20–35% of pectin (Mohnen 2008). In contrast to other pectin types, the backbone of RG-I consists of alternating α-1,2-linked l-rhamnose and α-1,4-linked d-galUA units. In addition, rhamnosyl residues in the RG-I backbone are often attached by side chains containing l-arabinose and d-galactose (Guillon et al. 1989; Colquhoun et al. 1994). Substituted HGs contain pectic heteropolymers with an α-1,4-linked d-galUA backbone attached to different side chains. They are classified on the basis of their side chain composition. Rhamnogalacturonan II (RG-II) is the most abundant of the substituted HGs, and it represents about 10% of pectin (O’Neill et al. 2004). Side chains in RG-II are composed of 12 different monomers including d-galactose, l-galactose, l-arabinose, l-rhamnose, d-xylose, l-fucose, d-apiose, d-galUA, d-glucuronate, 2-keto-3-deoxy-d-lyxo-heptulosaric acid (dha), 2-keto-3-deoxy-d-manno-octulosonic acid (kdo) and l-aceric acid (Mohnen 2008). Other substituted HGs include the less common heteropolysaccharides apiogalacturonan (AP) and xylogalacturonan (XGA), with d-apiofuranose or d-xylose side chains, respectively (Mohnen 2008).

Being abundant components of biomass, pectic polymers are degraded and the resulting monomers are catabolized by many bacterial and fungal microorganisms. Because the structure of pectin is very diverse, a complex set of pectin-degrading enzymes are produced and secreted by saprophytic microorganisms in order to release the monomers. These enzymes are also collectively referred to as pectic enzymes or pectinases. Pectic enzymes degrading the main chains of HG, RG-I or substituted HG are classified into hydrolases and lyases. Pectic hydrolases include exo- and endo-acting enzymes hydrolysing terminal d-galUA and l-rhamnose units at the non-reducing ends or internal bonds of pectic polymers, respectively (de Vries and Visser 2001; Culleton et al. 2013). For the pectic polymer XGA, specific xylogalacturonan hydrolases are active in the hydrolysis (van der Vlugt-Bergmans et al. 2000). In contrast to hydrolases, pectin-degrading lyases cleave the main chain through a β-elimination mechanism, forming unsaturated non-reducing ends (de Vries and Visser 2001). In addition to main chain degrading pectic enzymes, an extensive set of accessory enzymes, such as pectin methyl (Khanh et al. 1991) and acetyl (Searle-van Leeuwen et al. 1996) esterases, are needed for complete pectin degradation. Accessory enzymes including arabinofuranosidases, arabinases, galactanases, β-galactosidases, β-xylosidases, α-rhamnosidases and glucuronyl hydrolases are also required for complete pectin degradation (de Vries and Visser 2001).

Saprophytic filamentous fungi, such as *Aspergillus niger*, are capable of degrading pectic polymers. For example, 66 predicted genes that are possibly involved in pectin degradation are found in the genome of *A. niger* (Culleton et al. 2013), of which 46 were observed to be upregulated in the presence of monomeric pectin constituents or pectic polymers (Martens-Uzunova and Schaap 2009). In addition, *A. niger* is capable of growing on pectin-rich biomass (sugar beet and citrus pulp), pure pectin and many of the monomeric pectin constituents as sole carbon source (Fungal growth database). Pectin-degrading enzymes from fungi are also utilised e.g. in the beverage industry, where they are used to improve the juice yields and clarity of the final product.

## Microbial pathways for hexuronate catabolism

d-GalUA is catabolized by different life forms using different catabolic pathways. There are at least two prokaryotic and one eukaryotic pathway in microorganisms for the catabolism of d-galUA. The d-galUA and the d-glcUA pathways are similar in some life forms.

### Bacterial pathways for d-galacturonate and d-glucuronate

#### The bacterial isomerase pathways

**Fig. 2:**
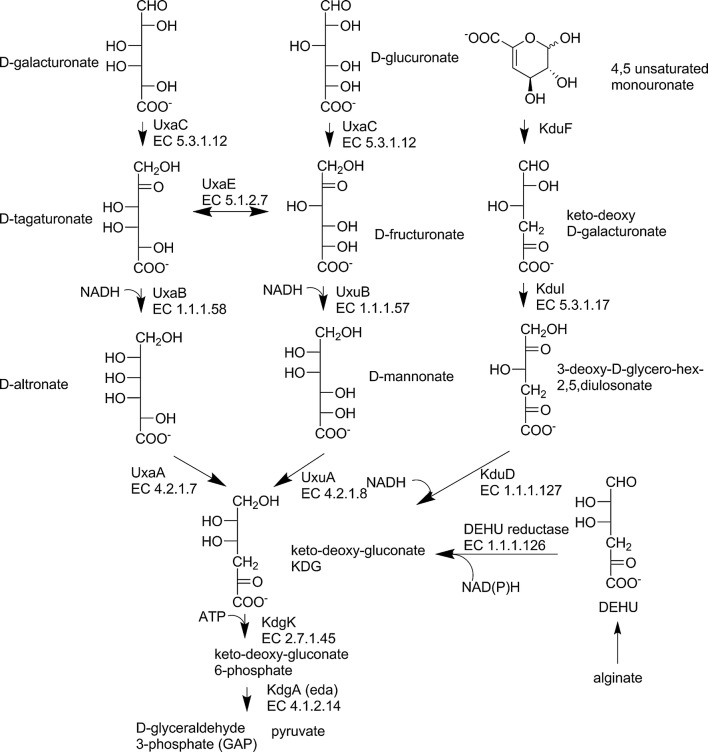
Bacterial pathways for hexuronate catabolism through keto-deoxy-gluconate, KDG. The pathways for d-galacturonate and d-glucuronate have the enzymes UxaC, KdgK and KdgA in common. The 4,5 unsaturated mono-uronate is the product of lyases acting on polygalacturonate, and DEHU (4-deoxy-l-erythro-5-hexoseulose uronate) is the product of lyases acting on alginate, a copolymer of two uronates, d-mannuronate and l-guluronate

In many bacterial species such as *Escherichia coli* and *Bacillus subtilis*, d-galUA is catabolized through the isomerase pathway with the genes *uxaC*, *uxaB*, *uxaA*, *kdgK* and *kdgA*. In this pathway, d-galUA is converted to pyruvate and d-glyceraldehyde 3-phosphate, GAP, at the expense of one NADH and one ATP. The *uxaC* codes for a uronate isomerase converting the d-galUA to d-tagaturonate. The *uxaB* codes for a d-tagaturonate reductase that forms d-altronate and has a specific requirement for NADH. The *uxaA* codes for a d-altronate dehydratase that forms 2-keto-3-deoxy gluconate, KDG. KDG is then catabolised to pyruvate and GAP by the enzymes KdgK and KdgA (Fig. 2). The *uxaC*, *uxaB*, *uxaA* and the *exuT* coding for a d-glcUA transport protein are organized in one operon (Mekjian et al. 1999).

d-GlcUA is catabolised using partly the same enzymes. The first enzyme from the d-galUA pathway, the UxaC, is also used in the first step of the d-glcUA pathway to form d-fructuronate. d-Fructuronate cannot be catabolized by UxaB and UxaA. Instead UxuB and UxuA convert d-fructuronate to d-mannonate and KDG. The UxuB is a d-fructuronate reductase requiring NADH and forming d-mannonate. UxuA is a mannonate dehydratase forming KDG, which is the same metabolite as in the d-galUA pathway. The enzymes for the reaction of KDG to KDG-6-phosphate and then to pyruvate and GAP, the KDG kinase, KdgK, and KDG-6-phosphate aldolase, KdgA, are used in the d-galUA and d-glcUA pathway. The genes *uxuB* and *uxuA* are in a separate operon, the genes *kdgK* and *kdgA* are not part of any of these operons. The *kdgA* is also called *eda* and is part of the Entner-Doudoroff pathway as well as of the d-galUA and d-glcUA pathways. The d-galUA and the d-glcUA pathways are different after the UxaC. In the hyperthermophilic bacterium *Thermotoga maritima*, an enzyme was identified that connects these pathways. This enzyme is a d-tagaturonate- d-fructuronate epimerase, UxaE (Fig. 2). With the aid of this enzyme d-galUA can be metabolized by UxuB and UxuA and d-glcUA by UxaB and UxaA (Rodionova et al. 2012).

The pathway for 5-keto-4-deoxy-galacturonate catabolism also has KDG as an intermediate. 5-Keto-4-deoxy-galacturonate is produced from pectin when lyases and not only hydrolases are degrading the pectin. 5-Keto-4-deoxy-galacturonate is converted by an isomerase, KduI, to 3-deoxy-d-glycero-hex-2,5-diulosonate, which is subsequently reduced to KDG by the NADH-dependentreductase KduD. This was described in *Erwinia chrysanthemi* (Condemine and Robert-Baudouy 1991). Homologous genes are also found in other bacteria such as *E. coli*. The KduD activity was demonstrated in *E. coli* (Hantz 1977). The *E. coli* enzymes KduI and KduD were however suggested also to have a role in hexuronate catabolism (Rothe et al. 2013), and KduD was shown to have activity with steroids (Tubeleviciute et al. 2014). Oligogalacturonate lyases produce 4,5-unsaturated mono-uronates, which were believed to spontaneously convert to the 5-keto-4-deoxy-galacturonate. This is however a slow process and an enzyme, KduF, was recently identified to catalyse this step (Hobbs et al. 2016) (Fig. 2). 5-Keto-4-deoxy-galacturonate can also originate directly from d-galUA by the action of a dehydratase. d-galUA dehydratases were described for *Microscilla* and *Geobacillus* (Groninger-Poe 2014).

#### The oxidative bacterial pathways

**Fig. 3:**
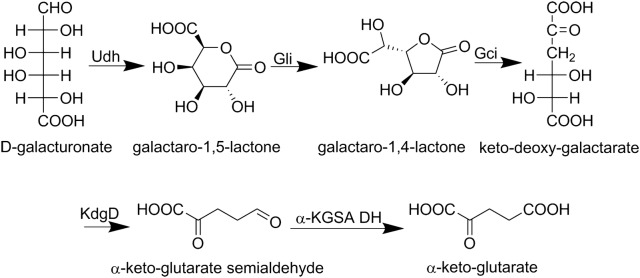
The bacterial pathway for oxidative galacturonate catabolism. Udh: d-galacturonate dehydrogenase EC 1.1.1.203; Gli: galactarolactone isomerase EC 5.4.1.4; Gci: galactarolactone cycloisomerase EC 5.5.1.27; KdgD: 5-dehydro-4-deoxy-glucarate dehydratase EC 4.2.1.41; α-KGSA DH: 2,5-dioxypentanate dehydrogenase EC 1.2.1.26

Some bacteria, such as *Agrobacterium tumefaciens* and *Pseudomonas species*, do not have the isomerase pathway but instead use an oxidative pathway (Fig. 3). In this pathway, d-galUA is converted to 2-ketoglutarate and CO_2_, with the reduction of two NAD(P)^+^. This pathway is best understood in *A. tumefaciens*. Here, the first enzyme is the uronate dehydrogenase (Udh), which reduces the d-galUA to galactarolactone using NAD^+^ as a cofactor. The galactarolactone in solution was identified as galactaro-1,4-lactone (Boer et al. 2010), however the crystal structure of the Udh revealed that it is the galactaro-1,5-lactone that is bound to the active site of the enzyme (Parkkinen et al. 2011). Although the conversion appeared to be spontaneous, a galactaro δ-isomerase (Gli) was identified, catalysing the reaction from the 1,5-lactone to the 1,4-lactone. The resulting 1,4-lactone was suggested to be the (2R, 3R, 4R, 5S) galactaro-1,4-lactone and not the (2S, 3S, 4S, 5R) isomer (Bouvier et al. 2014). The galactarolactone is then directly converted to the 2-keto-3-deoxy galactarate (3-deoxy-2-keto-l-threo-hexarate) by galactarolactone cycloisomerase (Gci) (Andberg et al. 2012). It was previously assumed that the galactarolactone would have been hydrolysed to galactarate in this pathway and that galactarate dehydratase produces the 2-keto-3-deoxy galactarate (Chang and Feingold 1970). At least in *A. tumefaciens* the shortcut catalysed by Gci is used. The next step is a combined dehydratase and decarboxylase reaction that results in 2-keto-glutarate semialdehyde. The 2-keto-glutarate semialdehyde is then oxidised by an NADP^+^-dependentdehydrogenase to 2-ketoglutarate. Genes for the last two enzymes were first identified in *Acinetobacter baylyi* (Aghaie et al. 2008). The enzymes of this pathway can be used for the catabolism of d-galUA and d-glcUA. The Udh is unspecific and produces galactarolactone or glucarolactone depending on the substrate. Whether or not the Gli is active with glucarolactone is unclear, but the Gci accepts both lactones as substrate and the reaction product 3-deoxy-2-keto-l-threo-hexarate is common to both pathways. Recently a novel variant of the oxidative pathway was identified. Two previously uncharacterized lactonases (UxuL and UxuF) were identified that catalyse the ring opening of galactaro-1,5-lactone and glucaro-1,5-lactone to galactarate and glucarate, respectively. The lactonases were not active with the 1,4-lactones (Bouvier et al. 2018).

### Pathway for l-guluronate, d-mannuronate and l-iduronate catabolism

Alginate is a polymer composed of l-guluronate and d-manuronate. The polymer is degraded by the action of alginate lyases, which results in the monomer 4-deoxy-l-erythro-5-hexoseulose, DEHU (Fig. 2). In *Pseudemonas* species DEHU is reduced by an NADPH-dependent reductase to 2-keto-3-deoxy-d-gluconate, KDG. KDG is then phosphorylated and subsequently split by an aldolase to pyruvate and glyceraldehyde 3-phosphate (Preiss and Ashwell 1962). The pathway has been expressed in the yeast *S. cerevisiae* (Enquist-Newman et al. 2014). The authors identified DEHU reductases from *Vibrio splendidus* and *Vibrio harveyi* that prefer NADH as a cofactor, and identified a DEHU transport protein from *Asteromyces cruciatus* that was active when expressed in yeast (Enquist-Newman et al. 2014).

l-iduronic acid is present in ulvan at a high level (Glasson et al. 2017). It is however not clear what microbial catabolic reactions are responsible for its catabolism.

### Fungal pathways

**Fig. 4:**
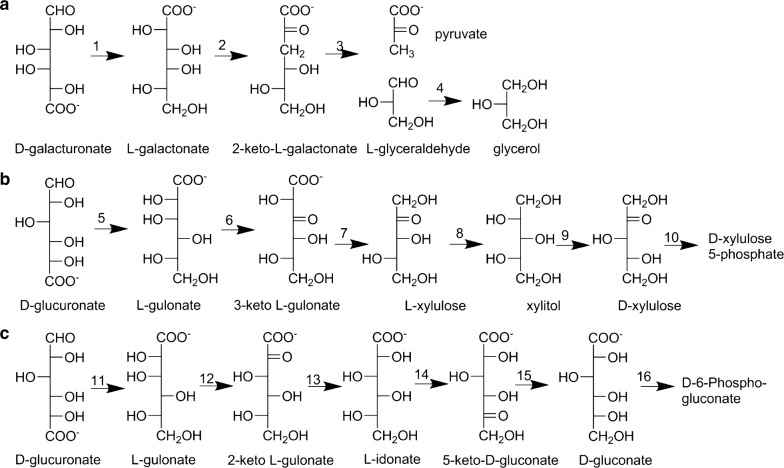
Eukaryotic pathways for d-galacturonate and d-glucuronate catabolism. **a** Fungal pathway for d-galacturonate catabolism: (1) d-galacturonate reductase EC 1.1.1.365 (2) l-galactonate dehydratase EC 4.2.1.146 (3) 2-keto-3-deoxy-galactonate aldolase EC 4.1.2.54 (4) l-glyceraldehyde reductase EC 1.1.1.372. **b** Animal pathway for d-glucuronate catabolism: (5) d-glucuronate reductase EC 1.1.1.19 (6) l-gulonate 3-dehydrogenase EC 1.1.1.45 (7) 3-dehydro-l-gulonate decarboxylase EC 4.1.1.34 (8) l-xylulose reductase EC 1.1.1.10 (9) Xylitol dehydrogenase EC 1.1.1.21 (10) xylulokinase EC 2.7.1.17. **c** Fungal pathway for d-glucuronate catabolism: (11) d-glucuronate reductase EC 1.1.1.19 (12) hypothetical reaction (13) 2-keto-l-gulonate reductase (14) l-idonate-5-dehydrogenase EC 1.1.1.264, EC 1.1.1.366 (15) 5-keto-gluconate reductase EC 1.1.1.69 (16) gluconokinase EC 2.7.1.12

The fungal pathway for d-galUA was first described in the mould *Trichoderma reesei*. In this pathway, d-galUA is converted to glycerol and pyruvate at the expense of two NADPH. It consists of an NADPH-dependent d-galUA reductase, Gar1 (Kuorelahti et al. 2005), an l-galactonate dehydratase, Lgd1 (Kuorelahti et al. 2006), a 2-keto-3-deoxy-l-galactonate aldolase, Lga1 (Hilditch et al. 2007) and an NADPH-dependent l-glyceraldehyde reductase, Gld1 (Liepins et al. 2006). The same pathway was also described in *Aspergillus niger* (Martens-Uzunova and Schaap 2008) and in *Botrytis cinerea* (Zhang et al. 2011) (Fig. 4a). In *A. niger,* the d-glaUA reductase, GaaA, is unspecific and can also accept NADH as a cofactor. d-GalUA reductases are also found in plants, where they have a role in l-ascorbate production. These enzymes are also NADPH-dependent (Agius et al. 2003), whereas an unspecific enzyme present in *Euglena gracilis* was described which also accepts NADH (Ishikawa et al. 2006).

A transcriptional activator for pectin and d-galUA catabolism, GaaR, was identified in *A. niger* (Alazi et al. 2016) and *B. cinerea* (Zhang et al. 2016b). A repressor of the GaaR, called GaaX, was identified in *A. niger*. Deletion of the *gaaX* gene resulted in strains that constitutively express GaaR-induced genes. The genes *gaaX* and *gaaR* are clustered and the clustering of these two genes is conserved in ascomycetes filamentous fungi (Niu et al. 2017). The compound that induces the transcription was identified as d-galUA or a compound derived from it (de Vries et al. 2002), and recently as 2-keto-3-deoxy-l-galactonate (Lin and Shaw 2007; Alazi et al. 2017).

### Animal pathway for d-glucuronate metabolism

d-GlcUA is a central metabolite in mammalian metabolism. It can be derived from UDP-glucuronate (Linster and Van Schaftingen 2006), from myo-inositol by the action of inositol oxygenase or from mucopolysaccharides (also referred to as glycosaminoglycans) through the action of α- or β-glucuronidases. d-GlcUA is catabolised through a pathway that is sometimes called the glucuronate-xylulose-pentose phosphate (GXPP) pathway (Hankes et al. 1969) or the uronate cycle. It was estimated that about 5% of glucose is metabolised through this pathway in mammals (Kaneko et al. 1997). The enzymes of this pathway are: d-glcUA reductase (EC 1.1.1.19) (Sato and Kador 1993), l-gulonate 3-dehydrogenase (EC 1.1.1.45) (Ishikura et al. 2005), 3-keto-l-gulonate decarboxylase (EC 4.1.1.34) (Smiley and Ashwell 1961) (Goode et al. 1996), l-xylulose reductase (EC 1.1.1.10) (Ishikura et al. 2001), xylitol dehydrogenase (EC 1.1.1.9) and xylulokinase (2.7.1.17). The last enzyme produces d-xylulose-5-phosphate, which is a metabolite in the pentose phosphate pathway (Fig. 4b).

### Fungal pathway for d-glucuronate

The fungal d-galUA reductases are unspecific for the substrate and also convert d-glcUA to l-gulonate. Indeed, GaaA is the first enzyme in the d-galUA as well as in the d-glcUA pathway. The other parts of the d-glcUA pathway are however completely different (Fig. 4). The l-galactonate dehydratase was specific for l-galactonate in *T. reesei* (Kuorelahti et al. 2006) and *A. niger* also does not appear to have an l-gulonate dehydratase (Motter et al. 2014). The fungal d-glucuronate pathway is not only different to the fungal d-galacturonate pathway, but is also different to the other eukaryotic path for d-glucuronate catabolism, the animal pathway. The fungal pathway for d-glcUA catabolism produces d-gluconate. The intermediates are l-gulonate, 2-keto-l-gulonate, l-idonate and 5-keto-d-gluconate. The genes and corresponding enzymes have been described in *A. niger* except for the reaction from l-gulonate to 2-keto-l-gulonate (Kuivanen et al. 2016a, 2017; Kuivanen and Richard 2018). In *A. niger,* d-gluconate is then phosphorylated to gluconate-6-phosphate, which is part of the pentose phosphate pathway (Müller 1985) (Fig. 4c). There is however also one report about an alternative pathway for d-gluconate in *A. niger* (Elzainy et al. 1973).

## Engineering hexuronate conversions in microbes

### d-Galacturonate uptake

When engineering microbes which do not naturally catabolise d-galUA for d-galUA conversions, the uptake of d-galUA must be addressed. In bacteria, the ExuT is responsible for transport of the hexuronates d-galUA and d-glcUA (Nemoz et al. 1976). In fungal microorganisms, other transporters are required. The yeast *Saccharomyces cerevisiae* was reported to take up d-galUA in low-pH conditions, although no protein for this transport activity could be identified (Souffriau et al. 2012). A d-galUA transport protein Gat1 was identified in *Neurospora crassa*. This protein was expressed in *S. cerevisiae*. Co-expression with a d-galUA dehydrogenase (Udh) or reductase (GaaA) resulted in the production of galactarate or l-galactonate, respectively, showing that the protein does indeed facilitate the transport of d-galUA to the cytosol (Benz et al. 2014). Deletion of the *gat1* gene in *N. crassa* resulted in reduced growth on pectin (Benz et al. 2014). Another transport protein was identified in *Botrytis cinerea.* In this case the deletion also affected growth on pectin (Zhang et al. 2014). Recently a GalUA transport protein from *A. niger*, GatA, was identified. When expressed in *S. cerevisiae* the GatA was about 50 times more active than the Gat1 and it was not inhibited by glucose in the medium (Protzko et al. 2018).

### Engineering microbes for ethanol production

d-GalUA is more oxidised than the sugars generally used for ethanol fermentation, which makes it challenging as a substrate for ethanol production. Using the bacterial isomerase pathway under anaerobic conditions would require two NADH per d-galUA to produce equimolar amounts of ethanol and CO_2_. It was suggested to express the isomerase pathway in yeast with the aim of using the resulting strain in co-fermentations with hexose and pentose sugars (van Maris et al. 2006). During the anaerobic sugar fermentation, a significant fraction of the carbon is directed to glycerol to compensate for oxidative, NADH-generating reactions in biosynthesis. The co-fermentation with d-galUA would provide an alternative NADH sink and would direct more carbon to ethanol (van Maris et al. 2006). Parts of the isomerase pathway have been expressed in *S. cerevisiae*: The genes *uxaC* and *uxaB* were expressed as active proteins (Huisjes et al. 2012), as well as kdgK and kdgA (Enquist-Newman et al. 2014). Expression of the complete bacterial isomerase pathway in yeast is still to be demonstrated.

In an alternative approach, the fungal d-galUA pathway, including a d-galUA transporter from *N. crassa*, was expressed in *S. cerevisiae*. All enzymes of the pathway were shown to be actively expressed; however, the resulting strain was not able to grow on d-galUA as the sole carbon source. Nevertheless, d-galUA was catabolized in a co-fermentation with a fermentable sugar (Biz et al. 2016). The low activity of the Lgd1 seemed to prevent growth on d-galUA. In the recent work of (Protzko et al. 2018) the Lgd1 was N-terminally tagged with a yellow fluorescence protein (Venus) resulting in a 60 fold increase in activity and resulting in growth on d-galUA when the complete pathway was expressed.

Hexuronates from alginate were fermented to ethanol using engineered *S. cerevisiae*. Alginate was hydrolysed with alginate lyase to generate the 2-keto-3-deoxy form of d-mannuronate and l-guluronate, DEHU. The strains contained a DEHU transporter and the DEHU pathway as shown in Fig. 2. To compensate for the NADH requirement, a mannitol pathway was expressed. The resulting strains were able to co-ferment mannitol and DEHU under anaerobic conditions to ethanol, achieving yields up to 83% of the maximum theoretical yield from the consumed sugars and titres of 36  g/l (Enquist-Newman et al. 2014).

### Engineering microbes for butanediol production

For the production of butanediol from d-galUA, the oxidative pathway as in Fig. 3 was used. The pathway was introduced to *E. coli* to produce 2-keto-glutarate semialdehyde (2,5-dioxopentanoate). This was then converted by a 2-keto acid decarboxylase (KDC) and an alcohol dehydrogenase to 1,4-butanediol, which was also expressed in *E. coli*. Since *E. coli* has endogenous pathways for d-galUA and galactarate catabolism, the *uxaC* and the *garD* coding for d-galUA isomerase and galactarate dehydratase, respectively, also had to be deleted. The resulting strain produced about 20 g/l butanediol from d-galUA (Tai et al. 2016).

### Engineering microbes for galactarate production

Galactarate is in itself a useful chemical. It is currently used in skin care products and was used as an acidifier in self-rising flour (Anonymus 1922). It is also the starting material for a chemical conversion to other useful chemicals, e.g. it can be quantitatively converted to adipic acid (Li et al. 2014), which is used for production of nylon.

d-GalUA acid can be oxidized to *meso*-galactarate in a microorganism expressing uronate dehydrogenase, udh. This was first demonstrated in the mould *T. reesei*. To prevent d-galUA catabolism, the endogenous catabolic pathway was disrupted by deleting the gene *gar1* encoding d-galUA reductase and overexpressing the bacterial d-galUA dehydrogenase *udh* (Mojzita et al. 2010). Later on, process optimization resulted in a galactarate titre up to 20 g/l from d-galUA (Barth and Wiebe 2017), and production from hydrolysed pectin with 18, 21 and 14 g/l galactarate titres in 1, 10 and 250 l bioreactor cultivations, respectively (Paasikallio et al. 2017). A more suitable mould for the pectin conversion was considered to be *A. niger*, since this mould produces pectinases efficiently. *A. niger* is however able to catabolise galactarate (Mojzita et al. 2010). Eliminating the galactarate catabolism was hampered by the fact that no fungal pathway had been described. To identify the genes of that pathway, mRNA of galactarate-grown mycelia was sequenced to identify the genes that were upregulated. These genes were then deleted and three genes coding for proteins with unknown function were identified to be essential for galactarate catabolism. Elimination of galactarate catabolism in combination with the expression of a udh resulted in a strain that was capable of producing galactarate not only from d-galUA, but also directly from pectin and CPW as a consolidated bioprocess (Kuivanen et al. 2016b).

A similar approach was made in *E. coli*. To prevent d-galUA and galactarate catabolism, the *uxaC* and *garD* genes were deleted and the d-galUA dehydrogenase *udh* was expressed. The resulting strain converted the d-galUA quantitatively to galactarate (Zhang et al. 2016a). In addition, galactarate production was demonstrated in an engineered *S. cerevisiae* strain containing the transport protein Gat1 and Udh (Benz et al. 2014).

### l-Galactonic and 2-keto-3-deoxy-l-galactonic acids

l-Galactonate (l-GalA), a metabolite of the fungal d-galUA pathway, is an l-sugar acid with potential use in some applications. For example, it can be converted to l-ascorbic acid, l-AA, also known as vitamin C, through biochemical (Roland et al. 1986) or chemical (Csiba et al. 1993) conversion. l-GalA can be produced from d-galUA through biochemical reduction of the aldehyde group at C1. This can be done using engineered strains of moulds having the disrupted l-galA dehydratase gene. The production from d-galUA has been demonstrated with engineered *H. jecorina* (*T. reesei*) and *A. niger* strains (Kuivanen et al. 2012). In bioreactor experiments, the highest product titre and yield observed with the *H. jecorina* strain from pure d-galUA were 7.2 g/l and 70% of the theoretical yield, whereas the *A. niger* performed less well in the same cultivation conditions (Kuivanen et al. 2012). However, low pH appears to favour l-galA production in *A. niger.* Similar titres of around 7 g/l were produced by the engineered *A. niger* strain cultivated in shake flasks when the pH was decreased to 3 (Kuivanen et al. 2012). With both organisms (*H. jecorina* and *A. niger*), the production required supplementation of a co-substrate (d-xylose) providing reducing power for the conversion. Consolidated bioprocessing from polygalacturonate (Kuivanen et al. 2012) and from citrus processing waste (Kuivanen et al. 2014) to l-galA has been demonstrated with the engineered *A. niger* strain. *A. niger* produces pectic enzymes and is capable of degrading pectin-rich biomass without significant biomass pretreatment steps (Kuivanen et al. 2014). In the case of polygalacturonate, around 8 g/l of l-galA was produced from the initial 15 g/l polygalacturonate in a bioreactor cultivation (Kuivanen et al. 2012). The consolidated bioprocess from CPW was carried out both as submerged and solid state fermentations in shake flasks and the product titres and yields were similar to those observed from pure d-galUA (Kuivanen et al. 2014). In addition to the engineered moulds, d-galUA conversion to l-galA has been demonstrated in engineered *S. cerevisiae* strains by expressing a heterologous d-galUA reductase from *A. niger* (Benz et al. 2014) or *Cryptococcus diffluens* (Matsubara et al. 2016).

2-Keto-3-deoxy-l-galactonic acid (2K3D-l-galA) is an intermediate of the fungal catabolic d-galUA pathway, but is also a keto-deoxy sugar with the potential of being used as a precursor for chemical synthesis. 2K3d-l-galA was produced using engineered *T. reesei* and *A. niger* strains (Wiebe et al. 2010). Deletion of the 2K3D-l-galA aldolase-encoding gene *lga1* from *T. reesei* resulted in a strain producing 10.5 g/l 2K3D-l-galA from 20 g/l d-galUA in a bioreactor. In addition, an *A. niger* strain with the deleted aldolase-encoding gene *gaaC* produced 15 g/l 2K3D-l-galA from 20 g/l polygalacturonate in a consolidated bioprocess.

### l-Ascorbic acid

l-AA is widely used as an antioxidant in the food, beverage and feed industries and as a supplement in pharmaceuticals, with the annual market volume exceeding 100,000 tons (Pappenberger and Hohmann 2014). Currently, the industrial production is mainly based on a multi-step process combining chemical and biotechnological steps, in which d-glucose is converted to l-AA (Pappenberger and Hohmann 2014). A purely biotechnological one-step process could be advantageous due to lower processing costs. Recently, a one-step biochemical process converting d-galUA to l-AA was demonstrated using an engineered *A. niger* strain (Kuivanen et al. 2015). As in the case of l-galA-producing *A. niger* strains, the native l-galA dehydratase activity was disrupted from the d-galUA pathway and two heterologous genes, encoding an l-galA lactonase and an l-galactono-1,4-lactone (l-galL) dehydrogenase (GALDH), both from a plant biosynthetic l-AA pathway, were expressed. In this pathway, l-galA is first lactonized to l-galL, followed by an oxidation reaction forming l-AA in the next step. As a result, low concentrations of l-AA, around 80 mg/l from pure d-galUA and 170 mg/l from CPW, were observed (Kuivanen et al. 2015). The majority of d-galUA in the process was converted to l-galA but not further to l-AA. Inefficiency of the lactonization reaction was considered to limit the process in the current strains (Kuivanen et al. 2015).

## Abbreviations

d-galUA: d-galacturonate
d-gluUA: d-glucuronate
l-galA: l-galactonate
l-AA: l-ascorbate
2K3D-l-galA: 2-keto-3-deoxy-l-galactonate
DEHU: 4-deoxy-l-erythro-5-hexoseulose
HG: homogalacturonan
XGA: xylogalacturonan
RGII: rhamnogalacturonan II
RGI: rhamnogalacturonan I
